# Zero-Waste Approach Applied to Pomegranates for Prolonging Fish Burger Shelf Life

**DOI:** 10.3390/foods11040551

**Published:** 2022-02-15

**Authors:** Olimpia Panza, Amalia Conte, Matteo Alessandro Del Nobile

**Affiliations:** Department of Agricultural Sciences, Food and Environment, University of Foggia, Via Napoli, 25, 71122 Foggia, Italy; olimpia.panza@unifg.it (O.P.); matteo.delnobile@unifg.it (M.A.D.N.)

**Keywords:** pomegranate, by-products, zero waste, fish shelf life, sustainability

## Abstract

In this study, the possibility of using whole pomegranate (juice, peel and seeds) according to the zero-waste approach, to prolong fresh fish shelf life, was evaluated. A preliminary antimicrobial in vitro test was carried out with peel and seeds as ground and re-ground powders. Then, the entire fruit, in the right proportions of juice and relative by-products as ground or re-ground powders, was added to fresh fish burger formulation to extend its shelf life. To this aim, a shelf-life test was performed on fortified fish products stored at 4 °C. Control samples were also tested for comparison. Specifically, the pH and microbiological and sensory quality of all the fish burgers were monitored during refrigerated storage for about 1 month. The results from the in vitro test clearly indicate that the peel is abundantly more effective than seeds on selected spoilage bacteria and that the ground peel powder is slightly more antimicrobial than the same re-ground powder. Results from the shelf-life test assessed that the control sample became unacceptable within a few days (about 3 days), while the samples with pomegranate juice and by-products maintained microbial stability for a longer time (2 or 3 weeks) (*p* < 0.05). The main microbiological problems are the proliferations of mesophilic and psychrotrophic bacteria, *Pseudomonas* spp. and *Shewanella.* The addition of pomegranate to the formulation allowed the fish spoilage to be controlled by at least 2 or 3 log cycles. In agreement with findings from the in vitro test, the best results from the microbiological point of view were found in fish burgers with juice, peel and seed ground powders. Furthermore, the addition of pomegranate was also appreciated from the sensorial point of view. In fact, products with pomegranate were prized for about 3 weeks for color, odor, appearance and texture of both raw and cooked products. Therefore, the current study reveals that the incorporation of the entire pomegranate, added in all parts according to the zero-waste concept, could promote a significant shelf-life extension of fish burgers, mainly due to the bioactive compounds present in fruit by-products, without changing the sensory quality.

## 1. Introduction

Fruits and vegetables are essential foods for human nutrition, but up to half of them (seed, peel, etc.) is thrown as waste—the so-called by-products—thereby, incurring significant costs and provoking serious environmental problems [1]. At the same time, by-products are also considered sources of valuable bioactive compounds with recognized nutritional properties and biological potential [1,2,3], being rich in polyphenols and flavonoids and, thus, playing an important role as natural antioxidant and/or antimicrobial agents [4,5]. For these reasons, by-products can be recycled to produce fortified products that are able to preserve and improve human health, with a view to a more sustainable approach [6]. In this perspective, also considering the growing interest in using natural additives to produce foods with healthful properties, numerous food commodities have been fortified with by-products or their extracts [7]. Furthermore, given that the antioxidant and the antimicrobial activity of compounds from by-products is comparable to that of synthetic preservatives [8,9], many times, their application was also aimed to prolong food shelf life [10].

In this context, the theory of “zero waste” is very interesting because it aims to develop adequate strategies and policies to manage all food waste and to limit their impact on the environment and society. Zero-waste manufacturing involves designing products and processes such that no trash is sent to landfills or incinerators because all the parts of a food, including the by-products, are completely utilized in the food formulation [11]. The increased use of entire fruit and vegetables will enhance the utility, versatility and economics of these crops. For this reason, some efforts have been made at lab-scale to utilize both the edible parts and the by-products of fruit and vegetables as added-value ingredients, with the potential of being processed for developing fortified food products. In this framework, some recent valid examples can be cited. Flesh and by-products from pumpkins were used for the manufacture of extrusion-cooked expanded foods [12]. Marinelli et al. [13] developed, with success, a watermelon-based jelly candy, without generating waste. Other authors [14,15,16] separately conducted a study on the full use of very ripe bananas as food ingredient for muffins, breads and wurstel. Romano et al. [17] analyzed the case of tomato puree obtained from two varieties of the whole fruit, including seeds and peels. 

Despite the potential of the total recycling of by-products according to the zero-waste concept, the use, at the same time, of both fruit juice and by-products to prolong fresh food shelf life seems still very unfeasible. To date, a unique case study was reported in the literature as an attempt to prolong food shelf life. It is the research of Dilucia et al. [18] who applied the pulp and peel of prickly pears according to the zero-waste approach, to preserve fish quality during refrigerated storage. 

Among the fruit by-products with recognized functionality, peel and seeds from pomegranates play a great part. Pomegranate (*Punica granatum* L.) is a deciduous shrub, native to Iran. It is one of the world’s oldest known fruits, widely grown in many tropical and subtropical countries, especially in temperate agro-climatic conditions, such as in Mediterranean regions, California and Asia. Total production is estimated to be around three million tons [19]. Since the ancient times pomegranate has been regarded as a “healing food” with numerous beneficial effects. Pomegranate peels comprise nearly a 50% portion of the total fruit weight [20]. The bioactive compounds are concentrated in the peel rather than in other parts of the fruit. Pomegranate peel is a rich source of tannins and other phenolic and flavonoid compounds, thus having a higher antioxidant capacity than the seeds and juice [21,22]. Extracts more than the peel itself have been characterized and applied to improve food quality [23] or prolong food shelf life [24]. However, the complete peel recycle is more desirable than its extract [25]. There are two other examples of peel recycling, not in the food sector but in animal science. Specifically, pomegranate peels were used as a meal dietary supplementation for broiler chickens to improve the antioxidant status and quality of breast meat [26,27]. The literature also reported papers on recycled seed powders or their extracts, with the antioxidant and antiradical properties of pomegranate seeds also being well recognized [28]. In this context, a study carried out on the powder of pomegranate seeds highlighted the improved quality of gluten-free bread enriched with these by-products in terms of physical characteristics, sensory properties and antioxidant activity [29]. Ayoubi et al. [30] incorporated powder from pomegranate seeds into cupcakes, thus enhancing the food’s nutritional quality. Valdés et al. [31] added seeds in fish-gelatin-based films to develop a bio-based active polymeric film that was found to be effective in protecting food from lipid oxidation. 

On the basis of the above considerations, the current study is quite justified. In fact, the global production of pomegranates, the partial attempts made to date to valorize by-products, the information still lacking about the use of pomegranate peel and seed powders to prolong food shelf life and the potential of zero-waste are all strategic factors to justify further research to explore the topic. The findings reported to date in the scientific literature greatly promote studies in this sector, with the aim of giving more detailed information about the real advantages to be derived in terms of shelf-life prolongation when the zero-waste approach is adopted. This aspect represents the novelty of the current paper because in this work, for the first time, the entire pomegranate as juice and by-products, in the correct proportion among them (according to the zero waste), were valorized to prolong fresh fish shelf life. To demonstrate the effectiveness of pomegranate addition, a shelf-life test was carried out on control and fortified fish products. To this aim, the pH, the growth of the main spoilage microorganisms and the sensory quality were monitored during a proper refrigerated storage period.

## 2. Materials and Methods

### 2.1. Schematic Overview of the Experimental Study

**Figure 1:**
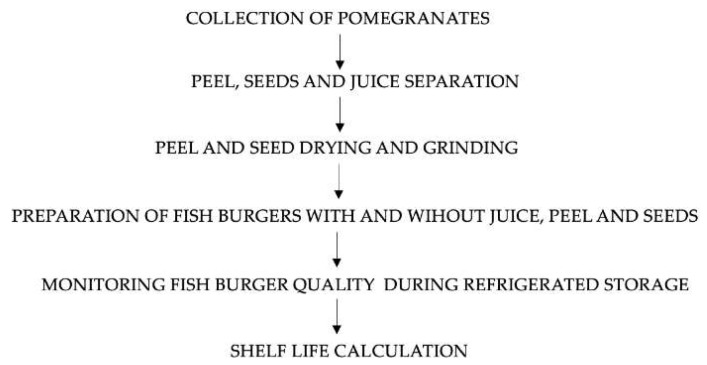
Schematic overview of the experimental plan.

A schematic overview of the experimental study is shown in Figure 1. Briefly, after the pomegranate collection, the peel, seeds and juice were separated. Before using, the peel and seeds were dried and ground into fine powders. Fish burgers with and without the three pomegranate parts were produced. To verify the effects of the pomegranates added to the fish formulation, a shelf-life test was carried out. 

### 2.2. Pomegranate Juice and By-Products

The pomegranates (*Punica granatum*, cv. Wonderful) were kindly provided by a local horticultural association (A.P.O. Foggia, Italy). The products were manually cut to separate the arils from the rest of the fruit. The pomegranate juice was obtained using a fruit domestic extractor (De’Longhi, Italy) and stored at −18 °C until use. The peel of the pomegranates was separated and cut into small pieces with a sharp knife. Then, the peel and seeds were dried in a ventilated oven at 38 °C for 48 h. The dried by-products were ground into powder in a laboratory blender and then sieved using a 500 µm filter sieve. Half of these powders were ground again to obtain finer powders (re-ground powders). Both the ground and the re-ground peel and seed powders were stored separately in plastic bags at −18 °C until use.

### 2.3. Fish Burger Preparation

For fish burger preparation, refrigerated salted cod fillets, potato flakes, potato starch and extra virgin olive oil, all purchased at local markets (Foggia, Italy), were used as basic recipe ingredients. Cod fillets were coarsely desalted, soaked and stored at 4 ± 1 °C for five days, changing the water every day. On the sixth day, the fish was drained to remove excess water for about half an hour, and the skin was removed. For the burger preparation, cod fillets were minced by a laboratory food processor (Fimar, Rimini, Italy). All the other above-reported ingredients were homogenized in a bowl (Multichef, Ariete, Firenze, Italy) equipped with a spiral hook for 5 min to obtain a homogeneous dough. To realize doughs with the addition of various pomegranate parts, the juice and the pomegranate powders from peel and seeds were added to the formulation. Three types of doughs were prepared, in addition to the control sample (named Ctrl = dough without pomegranate). Specifically, the first dough was named M1 and corresponded to the above fish formulation with a mix of juice and re-ground pomegranate by-products; the second dough named M2 corresponded to the above fish formulation with a mix of juice, water and re-ground pomegranate by-products; and finally, the third dough named M3, was the same as M1 but corresponded to the mix of juice and ground pomegranate by-products.

All the burger formulations are described in Table 1. As can be inferred from data reported in the table, the amounts of pomegranate juice and by-products in M1, M2 and M3 samples were defined following the criterion of the zero waste, i.e., the amounts of peel and seed powders, as fruit by-products added to the formulation, corresponded to the waste generated to obtain the amount of pomegranate juice mixed into the fish dough. In this way, the whole fruit was recycled in the fish burger without generating any further fruit waste. 

Fish burgers of 50 g were prepared by hand using a mini-burger mold (diameter 50 mm, thickness 10 mm); then, the samples were placed above a food tray with a pad and packaged in air using a high-barrier bag (multilayer film of nylon/polyethylene) with a thickness of 150 µm, provided by Biochemia (Bari, Italy) and kept under refrigeration (4 ± 1 °C) for about one month. 

On the raw samples, pH and microbiological quality were monitored, whereas, on both raw and cooked fish burgers, sensory properties were assessed, as described below in detail. 

### 2.4. Antimicrobial Activity of Pomegranate By-Products

The antimicrobial activity of pomegranate peel and seed ground and re-ground powders was evaluated by an in vitro test. To this aim, two strains of *Pseudomonas* spp. (*P. fluorescens* and *P. putida*) were used as target microorganisms and stored at −20 °C as stock cultures. The exponentially growing cultures were obtained in Plate Count Broth (PCB, tryptone 5 g/L, glucose 1 g/L and yeast extract 2.5 g/L, Oxoid) at 25 °C for 24 h. Subsequently, a cocktail of the two strains was diluted with 0.9% NaCl to obtain approximately 10^3^ CFU/mL. The PCB inoculated with the microbial cocktail was placed in several tubes. Every inoculated tube contained 3% of ground peel powder, 3% of ground seed powder, 3% of re-ground peel powder and 3% of re-ground seed powder for the active samples, respectively, and no powder for the control sample. All tubes were incubated at 25 °C for 72 h. Microbiological analyses were performed after 0, 24, 48, 72 and 120 h, taking aliquots of 1 mL from each tube. After appropriate dilutions with 0.9% NaCl, the samples were plated on *Pseudomonas* Agar Base (PAB, Oxoid), with an added cetrimide fucidin cephaloridine (CFC) selective supplement, incubated at 25 °C for 48 h. All analyses were performed in duplicate on two different samples.

### 2.5. Microbiological Analyses

During the entire storage period, both control and active samples were analyzed for microbial quality. To this aim, control samples and burgers with pomegranate (20 g each) were aseptically weighed into sterile stomacher bags, diluted with peptone water (dilution 1:10) and homogenized for 120 s with a Stomacher LAB Blender 400 (Pbi International, Milan, Italy). Subsequently, decimal dilutions of the homogenized samples were made using the same diluent, and the dilutions were plated onto specific media in Petri dishes to enumerate the following microbial groups: Plate Count Agar (PCA, Oxoid) incubated at 30 °C for 48 h and 5 °C for 10 days for mesophilic and psychrotrophic bacteria, respectively; *Pseudomonas* Agar Base (PAB, Oxoid), with an added cetrimide fucidin cephaloridine (CFC) selective supplement, incubated at 25 °C for 48 h for *Pseudomonas* spp.; Iron Agar (IA) supplemented with 5 g/L NaCl and incubated at 25 °C for 3 days, for hydrogen-sulfide-producing bacteria (HSPB); IA, supplemented with 10 g/L NaCl and incubated at 15 °C for 7 days, for psychrotolerant and heat-labile aerobic bacteria (PHAB); Violet Red Bile Glucose Agar (VRBGA, Oxoid) incubated at 37 °C for 24 h for Enterobacteriaceae. The microbiological analyses were conducted twice on two different samples. Results are expressed as log CFU/g. Microbial thresholds were set to 5 × 10^6^ CFU/g for total viable mesophilic and psychrotrophic bacteria, 10^6^ CFU/g for *Pseudomonas* spp. and *Shewanella* and 10^7^ CFU/g for *Photobacterium phosphoreum* [32]. The fitting of experimental data allowed the quantification of the microbiological acceptability limit (MAL), which represents the storage period (days) to reach the microbiological threshold. It was calculated according to the method used by Del Nobile et al. [33] in another study also dealing with fish burger shelf life. The goodness of fit was evaluated based on the χ^2^ value.

### 2.6. pH Determination

The measurement of pH was performed in triplicate on the first homogenized dilution of fish samples, using a pH meter (Crison, Barcelona, Spain). Two different samples were used for each measurement. 

### 2.7. Sensory Analyses

The quantitative descriptive analysis (QDA) was used for sample comparison, according to the guidelines of the Codex Alimentarius Commission. To this aim, fish burgers were submitted to a panel of five trained judges (aged from 30 to 45 years)—members of the laboratory with several years of experience in tasting food products before the current study. They were retrained for two days (1 session/day; 2 h/session) to establish the appropriate attributes for sensory evaluation, minimize individual differences and ensure repeatability of the results. The sensory evaluation was carried out both on raw and cooked burgers enriched with pomegranate juice and by-products. The samples were cooked at 200 °C for 20 min in an electric oven (Europa Forni, Vicenza, Italy). The panelists were asked to give judgments of raw and cooked samples in terms of odor, color, appearance and texture. In addition, they were also asked to give a global acceptance of raw and cooked burgers (overall quality). A nine-point scale was used to measure each attribute during the sensory evaluation. In the scale, 9 corresponded to excellent, 8 to very good, 7 to good, 6 to reasonable, 5 to the acceptable limit, 4 to dislike, 3 to bad, 2 to very bad and 1 to completely unacceptable [34]. Samples were differently coded and presented to each panelist simultaneously, in a random order. According to Del Nobile et al. [33], the fitting of the experimental data related to the overall quality allowed to quantify the sensory acceptability limit (SAL), which represents the storage period (days) to reach the sensory threshold (score = 5). The goodness of fit was evaluated based on the χ^2^ value.

### 2.8. Shelf-Life Calculation

As reported above, the experimental data were used for calculating both MAL and SAL values. Following Del Nobile et al. [33], these data were compared to assess the fish burgers’ shelf life. Briefly, the shelf life was considered the lowest value between the microbiological and the sensorial acceptability limits (MAL and SAL), indicating for both of them the periods of time during which the product remained acceptable. It was assumed the product was unacceptable when either one or both of them reached the threshold. 

### 2.9. Statistical Analysis

The fitting data were compared by a one-way analysis of variance (ANOVA). A Duncan’s multiple range test, with the option of homogeneous groups (*p* < 0.05), was carried out to determine significant differences among samples. STATISTICA 7.1 for Windows (StatSoft, Inc., Tulsa, OK, USA) was used.

## 3. Results and Discussion

### 3.1. In Vitro Antimicrobial Activity of Pomegranate By-Products

**Figure 2:**
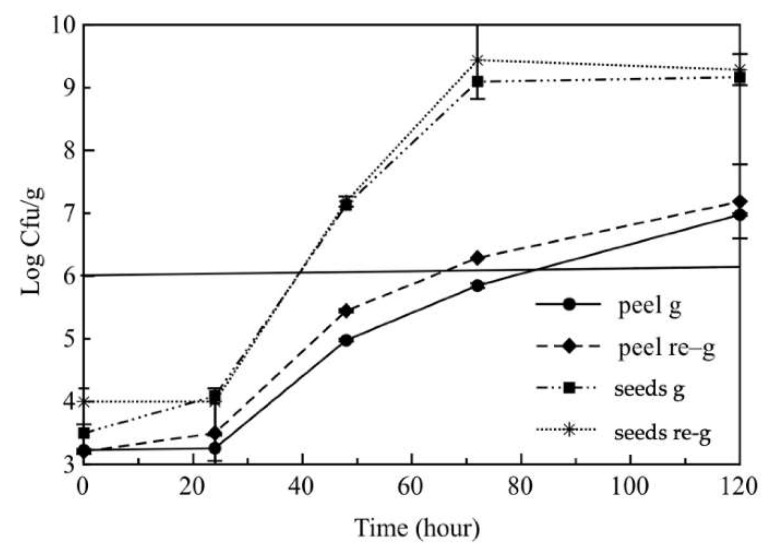
Evolution of *Pseudomonas* spp. viable cell concentration in broth with pomegranate ground peel powder (peel-g) and re-ground peel powder (peel re-g); ground seed powder (seed g) and re-ground seed powder (seed re-g).

A preliminary in vitro test was carried out on target bacteria, *Pseudomonas* spp., considering pomegranate peel and seed powders. The tests were run using both ground and re-ground powders. In this way, it was possible to compare the antimicrobial effects of the peel and seed; moreover, the effect of the double grinding on antimicrobial activity was also evaluated. The results are compared in Figure 2. In this figure, it is striking to observe a substantial difference between the peel and the seeds. Although in the first 24 h there was a very similar trend among samples, subsequently, a clear difference between the peel and the seeds was observed. The growth of *Pseudomonas* spp. was considerably delayed in the case of the peel and reached the microbial threshold within 72 h compared to the case for the seeds, in which the limit was reached just after 36 h. Data shown in Figure 2 clearly indicate that the peel is much more effective than the seeds against the investigated target microorganisms. This finding confirms data reported in the literature that also prove the capacity of fruit peel to control spoilage proliferation [21,22].

Comparing the results obtained between ground and re-ground samples, even though no relevant differences were observed, a slightly better antimicrobial effect was observed for the ground powders. Probably, this is due to the fact, that the second grinding process may have slightly compromised the powder’s efficacy because of the temperature rise during the grinding process. In the subsequent step of the study, both types of powders (ground and re-ground) were investigated.

### 3.2. Microbial Quality Decay of Fish Burgers

The microbial quality decay of the investigated fish burgers was determined by monitoring the viable cell concentration of total mesophilic and psychrotrophic bacteria, *Pseudomonas* spp., *S. putrefaciens*, *Photobacterium phosphoreum* and Enterobacteriaceae. 

**Figure 3:**
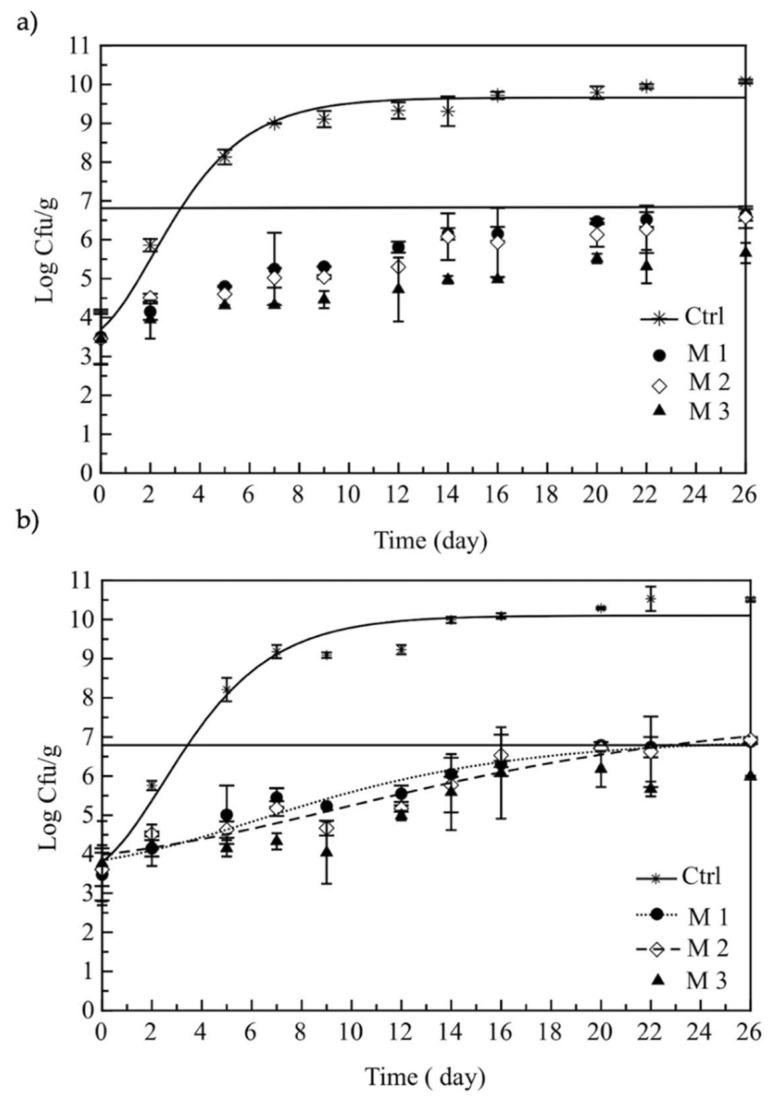
Evolution of total mesophilic (**a**) and psychrotrophic (**b**) bacteria in fish burgers during storage at 4 °C. Data are presented as mean ± SD. Symbols: experimental data; solid line: threshold for microbial acceptability set to 5 × 10^6^ CFU/g. Ctrl: fish burger; M1: fish burger with mix of juice and re-ground pomegranate powders; M2: fish burger with mix of juice, water and re-ground pomegranate powders; M3: fish burger with mix of juice and ground pomegranate powders.

Figure 3 shows the viable cell concentration of mesophiles (a) and psychrotrophs (b) for all the investigated samples, whereas the horizontal solid line is the microbial threshold. As can be inferred from data in both graphs, for these two microbial groups, the initial bacterial load was approximately 3.5 log CFU/g. In relation to mesophilic bacteria (Figure 3a), the control sample quickly reached the microbial limit, on the third day of storage itself; contrarily, all the samples with pomegranate never reached the threshold during the entire observation period (*p* < 0.05). It is well recognized that refrigerated and cold stored fishery products undergo significant enzymatic and chemical autolytic reactions, and therefore, when microbial load is exceeded, sea food quality is also compromised in terms of trimethylamine, total volatile basic nitrogen and lipid oxidation [35]. For this reason, the preservation of sea food with natural compounds from the edible parts of fruits and their by-products directly added to fish formulation could be strategic to control the numerous detrimental phenomena occurring in the fresh fish matrix. 

A similar trend was recorded also for the psychrotrophs (Figure 3b), but in this case, the threshold was reached after about 20 days for M1 and M2 samples, whereas it was never reached by the M3 sample, where the viable cell concentration remained always below 5 × 10^6^ CFU/g. Therefore, these results clearly show that the incorporation of pomegranate into the burger formulation, regardless of the grinding process, exerted a significant inhibitory action against total bacterial count compared to the control sample. Our results agree with the data from the scientific literature also dealing with pomegranate by-products. In particular, Zhuang et al. [24] also assessed with success the effects of pomegranate peel extract on the quality of bighead carp fillets during chilled storage. Incoronato et al. [36] also observed that the addition of pomegranate juice and by-products to pancake greatly contributed to delay the total microbial count’s proliferation. 

In regard to differences among the three samples with pomegranates, it can be highlighted that the M3 fish burger was the most effective against both the abovementioned spoilage microorganisms (*p* < 0.05). This evidence is in line with the preliminary test carried out with the sole powders where the ground peel and seeds were found to be more effective than the corresponding re-ground samples.

**Figure 4:**
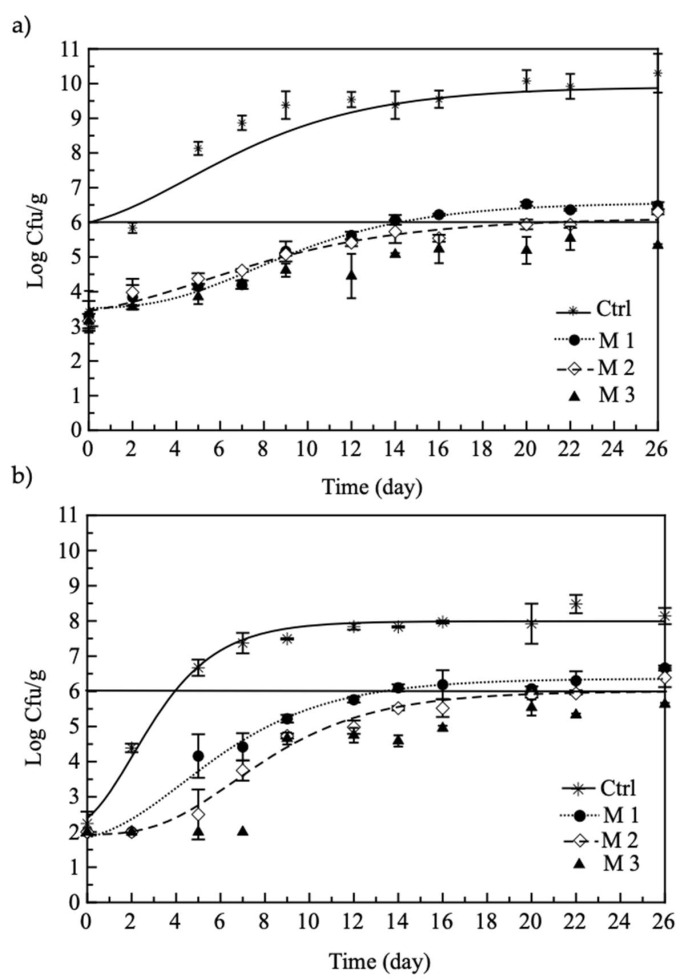
Evolution of *Pseudomonas* spp. (**a**) and *S. putrefaciens* (**b**) in fish burgers during storage at 4 °C. Data are presented as mean ± SD. Symbols: experimental data; solid line: threshold for microbial acceptability set to 106 CFU/g. Ctrl: fish burger; M1: fish burger with mix of juice and re-ground pomegranate powders; M2: fish burger with mix of juice, water and re-ground pomegranate powders; M3: fish burger with mix of juice and ground pomegranate powders.

Concerning specific fish-spoilage microorganisms, in Figure 4, the *Pseudomonas* spp. and *S. putrefaciens* viable cell concentration is plotted as a function of storage time. Compared to the control sample, the burgers with pomegranate show a different behavior. Specifically, the initial microbial load detected for the acitve samples was lower than that observed for the control sample (approximately 3 and 2 log CFU/g, for *Pseudomonas* spp. and *S. putrefaciens*, respectively). In addition, for the abovementioned microbial species, faster growth was found in the control burger in comparison to the other fish products. As a consequence, the control sample became unacceptable within a few days, whereas the burger loaded with pomegranate remained acceptable for a longer time, with slight differences among them. These data confirm the effectivenes of pomegranate also in reducing the growth of specific fish-spoilage microorganisms. [37]. Similar results have been reported in the literature by Alexandre et al. [38], who suggested that the antibacterial activity of pomegranate peel extracts may be related to the presence of polyphenols, tannins, flavonoids and anthocyanins that can alter the cell wall protein structure by disrupting the coaggregation of microorganisms, thus slowing down the microbial growth.

Comparing the samples loaded with pomegranate (i.e., M1, M2 and M3), some differences among them were observed. In particular, M1 reached the threshold value (10^6^ CFU/g) after about 14 days and M2 after about 21 days, whereas M3 never reached the threshold value during the entire storage period.

To highlight the differences among the investigated samples, in Table 2, the final concentrations (after 26 days) of *Photobaterium phosphoreum* and Enterobacteriaceae are reported. As can be seen, a significant difference was found between the Ctrl and the samples with pomegranate (*p* < 0.05). Although at the beginning of the storage all samples showed very low initial counts (approximately 2 log CFU/g) (data not shown), after a few days, the control reached the threshold (10^7^ CFU/g), whereas, in all the other samples, the cell loads remained always very low, till the end of storage.

A similar microbial proliferation on cod fish was also reported by Dilucia et al. [18], who added various concentrations of prickly pear pulp and peel to fish burgers. These authors also observed a reduction of the microbial growth rate, due to a proper concentration of prickly pear added to fish products, compared to samples without any addition. In a few cases, the growth rate was close to zero (i.e., total mesophilic bacteria and HSPB), and in one case (i.e., Enterobacteriaceae), a decrease in the viable cell concentration (negative growth rate) during refrigerated storage was observed. 

Looking at all the microbiological experimental data recorded in the current study, the control sample became microbially unacceptable in a few days, whereas the viable cell concentration of the samples with pomegranates remained under the microbial threshold for a much longer time, and in some cases, the threshold was never reached during storage.

To calculate the microbial acceptability limit (MAL), the fitting of the experimental data was carried out; the recorded fitting parameters are listed in Table 3. As can be seen from Table 3, MAL values differed significantly (*p* < 0.05) between control and fish burgers with pomegranates. Very low values were observed for the control sample, which became unacceptable in a few days. On the other hand, higher MAL values were observed for all the fish products loaded with juice and by-products (*p* < 0.05). Some difference was observed among these last three samples also. In fact, M1 remained acceptable for about two weeks and M2 for about three weeks, whereas M3 remained acceptable for the entire observation period (i.e., 26 days).

### 3.3. Fish Burger pH Evolution

**Figure 5:**
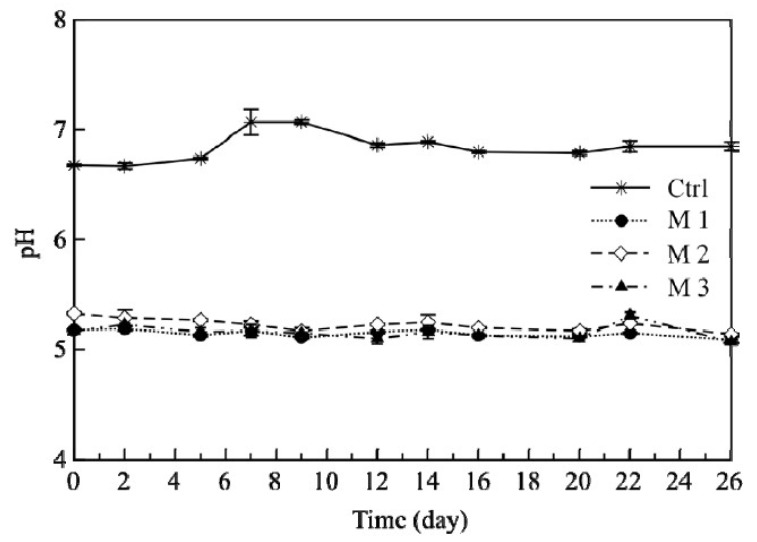
Trend of pH values of fish burgers during 26 days of storage at 4 °C. Data indicate means ± SD. Ctrl: fish burger; M1: fish burger with mix of juice and re-ground pomegranate by-products; M2: fish burger with mix of juice, water and re-ground pomegranate by-products; M3: fish burger with mix of juice and ground pomegranate by-products.

Figure 5 reports the time course during storage of the pH of the tested samples. From data shown in this figure, it is clear that even from the beginning of the test, the samples with pomegranates had lower values of pH (about 5.2) than the control fish samples (about 6.7) (*p* < 0.05). Data reported in this figure also highlight that the above difference among samples remained constant throughout the entire storage period. This difference was due to the direct addition of juice and pomegranate powders in the fish formulation that increased the content of tannins and phenolic acid of the food mixture, thus promoting a consequent pH reduction. Similar effects on pH were also recorded by Incoronato et al. [36], who also added pomegranate fruit in pancake and observed a reduction in the pH. In this study, it was also found that the pH remained lower during the entire storage. The lower pH is in agreement with the microbiological findings. In fact, the addition of pomegranate in terms of juice and by-products was able to delay the microbial proliferation of all spoilage groups due to the antibacterial activity of tannins and phenolic acid, as these compounds are also responsible for pH reduction [39]. 

### 3.4. Sensory Quality of Fish Burgers

The results from sensory evaluation of the investigated raw samples during refrigerated storage are reported in Table 4. As can be seen, only samples with pomegranate peel, seeds and juice were subjected to sensory evaluation because the control fish samples became unacceptable very quickly due to undesired microbial proliferation, and therefore, their sensory quality was not assessed. From Table 4, it can be inferred that all these samples showed a similar trend. In fact, the overall quality of all food products was very high at the beginning of the storage, with values around 9. As expected, over time, their appreciation dropped off. However, all these burgers remained above the threshold (score = 5) for about three weeks. The general trend found for the overall quality was in accordance with that of the specific sensory attributes used to evaluate fish quality (color, odor, appearance and texture—data not shown). The raw fish products were greatly appreciated for their slight pomegranate smell and the general rosy color. The fact that the burgers’ overall quality was found to be above the threshold for more than three weeks can be attributed to the addition of pomegranate fruit because bioactive compounds from the juice and by-products preserved the matrix from the microbiological spoilage and slowed down its sensory deterioration [24,36,40]. 

**Figure 6:**
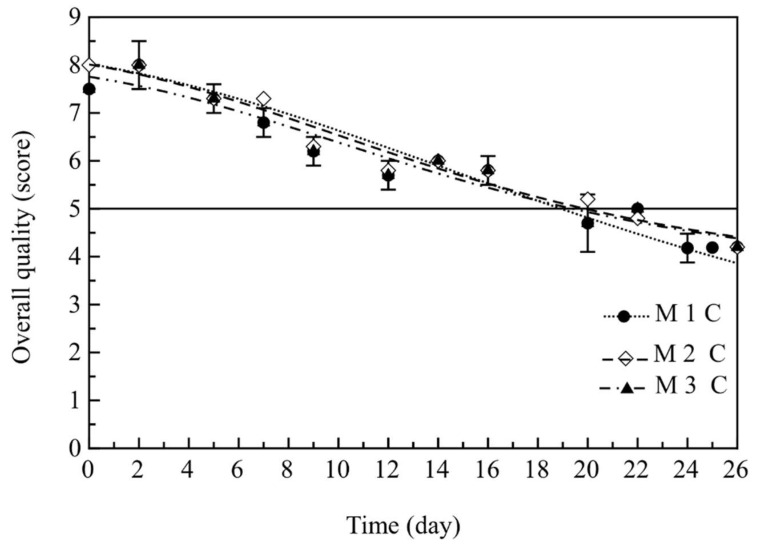
Trend of overall quality of cooked fish burgers during 26 days of storage at 4 °C. Data indicate means ± SD. M1 C: cooked fish burger with mix of juice and re-ground pomegranate by-products; M2 C: cooked fish burger with mix of juice, water and re-ground pomegranate by-products; M3 C: cooked fish burger with mix of juice and ground pomegranate by-products.

In Figure 6, the evolution during refrigerated storage of the cooked fish’s overall quality is reported. As can be inferred from data shown in the figure, a similar trend was observed for all the samples. No statistically significant difference among them was observed (*p* > 0.05), thus suggesting that the selection of the ground or re-ground powder did not influenced the overall quality. All these burgers containing pomegranate were first highly appreciated, and during storage, the panel found an increasing number of sensory defects, untill they became unacceptable. Data reported in Figure 6 agreed with results found for the specific sensory attributes (data not shown). In fact, samples were found acceptable for odor, color, appearance and texture for about three weeks. The sensory parameter that mostly influenced the overall quality was the texture, due to the intrinsic quality decay of fresh fish and in particular its drip loss [33]. Considering these findings, it is possibile to assess that pomegranate addition to fish burgers, in the form of juice and relative by-products, allowed the mantainence of product quality for about three weeks. 

In order to better highlight differences among samples, the re-parameterized Gompertz equation was used to fit the cooked burgers’ overall quality data (i.e., the overall quality plotted as a function of storage time) to calcualte the SAL values [33]. The results of the fitting procedure are listed in Table 3. As can be seen, there were only slight differences among the three samples M1, M2 and M3 (*p* > 0.05). In particular, it was found that M2 and M3 were more stable than M1 from the sensory point of view. However, it must be highlighted that these differences were not statistically significant (*p* > 0.05). 

### 3.5. Shelf Life of Fish Burgers

Comparing all data in Table 3 in terms of MAL and SAL, it is possibile to calculate the shelf life of each product as the lowest values reported in each row. Comparing the times during which each product remained acceptable from both the microbiological and the sensory point of view, it is possible to infer the final shelf life. Looking at data reported in the first row of Table 3, it is easy to see that the shelf life of control sample was linked to the *Pseudomonas* spp. contamination that reached 10^6^ CFU/g in a few days. Therefore, the control fish burger recorded a shelf life of less than 3 days. Different results were found for the other three samples with pomegranates (*p* < 0.05). In particular, the shelf life was less than two weeks for M1 and less than three weeks for M2 and M3 samples. It is clear that the M1 sample became unacceptable due to undesired *Pseudomonas* spp. proliferation after 14 days, whereas, the other two samples, M2 and M3, became unacceptable because sensory defects appeared on the products after about three weeks of monitoring. 

Therefore, the shelf-life prolongation of the three fish burgers with pomegranate, compared to control samples, was abundantly evident, and it was due to the addition of pomegranate to the formulation, which allowed the delay of both microbial and sensory decay. Some differences were also recorded among these samples, even though, in this case, it is not very easy to understand the reasons for the experimental differences because few data are available in the literature to make a proper comparison. Further study is still necessary for a deeper investigation of the pomegranate powders and their properties against the specific detrimental phenomena involved in fish decay. The interesting findings recorded from this preliminary study give reasons to continue the investigation because shelf-life prolongation from the perspective of a more sustainable approach, such as the zero-waste one, could represent the future challenge of the food industry.

## 4. Conclusions

In this study, the zero-waste approach was adopted with success by adding to a fish burger all parts of the pomegranate fruit, according to a proper combination between juice and relative by-products in the form of powders. The results showed that all fortified burgers achieved both microbiological and sensory improvements compared to the control. In particular, it was clearly noted that the control sample became unacceptable for microbial proliferation within a few days, while the other samples remained acceptable for about 2 or 3 weeks, depending on the type of powder addition (ground or re-ground). Moreover, all the fortified burgers were highly prized for their sensory properties. Therefore, considering both the microbial and sensory enhancements recorded with pomegranate addition to fish formulation, it is possible to assess that both fruit juice and by-products in a sustainable way can be used to prolong product shelf life. Considering this experimental evidence, the approach is interesting. Certainly, more in-depth research is necessary to further explore the topic and to elucidate how the zero-waste approach can serve as formidable candidate to reduce environmental impact. In particular, a proper comparison is recommended between the energy costs and emissions linked to the zero-waste approach and those associated with by-product disposal. In addition, it is necessary to identify the best practice that guarantees by-products as food-grade ingredients and to identify methods for measuring their safety level. 

## Figures and Tables

**Table 1:** Formulation of cod fish burgers with and without pomegranate addition.

Ingredients	M1 (g)	M2 (g)	M3 (g)	Ctrl (g)
Cod fish	50	50	50	50
Pomegranate juice	9.5	8.6	9.5	-
Water	-	0.9	-	-
Pomegranate by-products (peel)	9.14	8.14	9.14	-
Pomegranate by-products (seeds)	2.96	2.63	2.96	-
Potato starch	9.61	9.61	9.61	9.61
Extra virgin olive oil	9.61	9.61	9.61	5
Potato flakes	7.7	7.7	7.7	7.7

**Table 2:** Microbial counts (log CFU/g) of fish burgers after 26 days of storage.

Sample	*Photobacterium phosphoreum*	Enterobacteriaceae
Ctrl	7.25 ± 0.36 ^a^	7.95 ± 0.08
M1	3.74 ± 0.37 ^b^	2.00 ± 0.00 ^b^
M2	3.64 ± 0.90 ^b^	2.00 ± 0.00 ^b^
M3	3.00 ± 0.00 ^b^	2.00 ± 0.00 ^b^

Mean values ± SD. Means in the same column followed by different superscript letters are significantly different (*p* ˂ 0.05). M1: fish burger with mix of juice and re-ground pomegranate powders; M2: fish burger with mix of juice, water and re-ground pomegranate powders; M3: fish burger with mix of juice and ground pomegranate powders.

**Table 3:** Microbial acceptability limit (MAL), sensory acceptability limit (SAL) and shelf life of fish burgers.

Samples	Microbiological Acceptability Limit (Day)				Sensory Acceptability Limit (Day)	Shelf Life (Day)
	MAL^TMB^	MAL^TPB^	MAL*^Pse^*	MAL*^Shew^*	SAL^Cooked^	
Ctrl	3.12 ± 0.23 (χ^2^ = 0.74)	3.33 ± 0.30 ^a^ (χ^2^ = 0.49)	2.5 ± 0.20 ^a^ (χ^2^ = 0.88)	3.94 ± 0.25 ^a^ (χ^2^ = 0.48)	-	2.5 ± 0.20 ^a^
M1	>26	19.91 ± 0.99 ^b^ (χ^2^ = 0.65)	14.17 ± 0.85 ^b^ (χ^2^ = 0.28)	13.58 ± 0.97 ^b^ (χ^2^ = 0.55)	18.93 ± 0.87 ^a^ (χ^2^ = 0.86)	13.58 ± 0.97 ^b^
M2	>26	21.40 ± 0.92 ^b^ (χ^2^ = 0.98)	21.27 ± 4.10 ^c^ (χ^2^ = 0.30)	20.26 ± 2.44 ^c^ (χ^2^ = 0.68)	19.51 ± 0.83 ^a^ (χ^2^ = 0.69)	19.51 ± 0.83 ^c^
M3	>26	>26	>26	>26	19.01 ± 0.95 ^a^ (χ^2^ = 0.98)	19.01 ± 0.95 ^c^

Mean values ± standard deviation and χ^2^. Data in the same column followed by different superscript letters are significantly different (*p* < 0.05). M1: fish burger with mix of juice and re-ground pomegranate powders; M2: fish burger with mix of juice, water and re-ground pomegranate powders; M3: fish burger with mix of juice and ground pomegranate powders.

**Table 4:** Overall quality of raw fish burgers during storage at 4 °C.

Samples	Time (Day)										
	0	2	5	7	9	12	14	16	20	22	26
M1 Raw	9.0 ± 0.0 ^a^	7.5 ± 0.0 ^a^	7.5 ± 0.0 ^a^	6.5 ± 0.5 ^a^	6.3 ± 0.3 ^a^	6.0 ± 0.5 ^a^	5.8 ± 0.3 ^a^	5.7 ± 0.6 ^a^	4.5 ± 0.5 ^a^	4.3 ± 0.6 ^a^	4.0 ± 0.5 ^a^
M2 Raw	9.0 ± 0.0 ^a^	8.3 ± 0.3 ^b^	7.0 ± 0.0 ^a^	6.7 ± 0.3 ^a^	6.2 ± 0.3 ^a^	6.0 ± 0.0 ^a^	5.7 ± 0.3 ^a^	5.5 ± 0.0 ^a^	5.2 ± 0.3 ^b^	4.8 ± 0.3 ^a^	4.2 ± 0.3 ^a^
M3 Raw	9.0 ± 0.0 ^a^	8.2 ± 0.3 ^b^	7.0 ± 0.0 ^a^	6.3 ± 0.3 ^a^	5.8 ± 0.3 ^a^	5.7 ± 0.3 ^a^	5.5 ± 0.0 ^a^	5.5 ± 0.0 ^a^	5.0 ± 0.0 ^b^	5.0 ± 0.0 ^a^	4.2 ± 0.3 ^a^

Mean values ± standard deviation. Means in the same column followed by different superscript letters are significantly different (*p* < 0.05). M1: fish burger with mix of juice and re-ground pomegranate powders; M2: fish burger with mix of juice, water and re-ground pomegranate powders; M3: fish burger with mix of juice and ground pomegranate powders.

## Data Availability

Data presented in this study are available on request.
